# Seroprevalence of and Risk Factors for *Toxoplasma gondii* Infection in Cats from Greece

**DOI:** 10.3390/ani13071173

**Published:** 2023-03-26

**Authors:** Kassiopi Christina G. Kokkinaki, Manolis N. Saridomichelakis, Mathios E. Mylonakis, Leonidas Leontides, Panagiotis G. Xenoulis

**Affiliations:** 1 Clinic of Medicine, Faculty of Veterinary Science, University of Thessaly, 224 Trikalon Str., GR-43132 Karditsa, Greece; 2 Companion Animal Clinic, School of Veterinary Medicine, Aristotle University of Thessaloniki, 11 Stavrou Voutyra Str., GR-54627 Thessaloniki, Greece; 3 Laboratory of Epidemiology, Biostatistics and Animal Health Economics, Faculty of Veterinary Science, University of Thessaly, 224 Trikalon Str., GR-43132 Karditsa, Greece; 4 Gastrointestinal Laboratory, Department of Small Animal Clinical Sciences, College of Veterinary Medicine and Biomedical Sciences, Texas A & M University, College Station, TX 77843, USA

**Keywords:** feline, prevalence, protozoan, public health, seropositivity

## Abstract

**Simple Summary:**

Toxoplasmosis is one of the most important protozoan diseases with a global impact on the health of domestic cats and with zoonotic significance. A total of 457 cats were prospectively enrolled, and a commercially available indirect immunofluorescence antibody testing kit was used for the detection of anti-*Toxoplasma gondii* immunoglobulin G in serum. Overall, 95 (20.8%) of the 457 cats were seropositive for *T. gondii.* The results of this study indicate that older cats and cats with a history of cat-fight trauma are at the greatest risk for *T. gondii*-seropositivity. Furthermore, lack of vaccination against calicivirus, herpesvirus-1, panleukopenia, and rabies was identified as a risk factor for *T. gondii* seropositivity. This study shows a high prevalence of seropositivity for *T. gondii* in cats in Greece. This implies that toxoplasmosis is still a major public health concern and that optimal strategies for the prevention of infection with *T. gondii* in cats should be established.

**Abstract:**

Toxoplasmosis is one of the most important protozoan diseases with a global impact on the health of domestic cats and with zoonotic significance. The aims of this study were to determine the prevalence of seropositivity for *Toxoplasma gondii* in different populations of cats in Greece and to assess risk factors for seropositivity. A total of 457 cats were prospectively enrolled, and a commercially available indirect immunofluorescence antibody testing (IFAT) kit was used for the detection of anti-*T. gondii* immunoglobulin G (IgG) in serum. Overall, 95 (20.8%) of the 457 cats were seropositive for *T. gondii.* Based on multivariate analysis, factors associated with seropositivity included older age [Odds ratio (OR), 1.33; *p* < 0.001]; a history of cat-fight trauma (OR, 3.88; *p* = 0.004); and lack of vaccination against calicivirus, herpesvirus-1, panleukopenia, and rabies (OR, 10; *p* = 0.002). This study shows a high prevalence of seropositivity for *T. gondii* in cats in Greece. This implies that toxoplasmosis is still a major public health concern and that optimal strategies for the prevention of infection with *T. gondii* in cats should be established.

## 1. Introduction 

Toxoplasmosis is one of the most important protozoan diseases with a global impact on the health of domestic cats and is also of zoonotic importance [1]. The causative agent is *Toxoplasma gondii*, which infects virtually all species of warm-blooded animals (intermediate hosts). Domestic cats and other felids are the definitive hosts that excrete oocysts [2].

The main modes of transmission, in both intermediate and definitive hosts, are congenital infection through the placenta, ingestion of oocyst-contaminated food or water, and ingestion of infected raw tissue, with the last being the primary source of infection in cats [3]. Other, less important, modes of transmission include blood transfusion, lactation, and transplantation of tissues and organs [4].

During the life cycle of *T. gondii*, three developmental stages can infect cells: sporozoites, tachyzoites, and bradyzoites. Most cats are infected by ingesting intermediated hosts and shed resistant oocysts with their feces. Once passed to the environment, sporulation occurs in 1–5 days, and the sporulated (infectious) oocysts can survive for months to years [5]. After the primary infection of a cat, subsequent reinfections do not usually result in oocyst shedding, and even if this occurs, the load of shed oocysts is low [6,7,8,9].

The extra-intestinal life cycle of *T. gondii* may occur after the ingestion of tissue cysts or oocysts. After the exposure, sporozoites excyst in the lumen and become tachyzoites through an asexual process. Tachyzoites multiply intracellularly in almost any cell of the body and eventually encyst. The ensuing tissue cysts contain numerous bradyzoites.

Feline infection with *T. gondii* is usually subclinical, although vertical toxoplasmosis or infection of immunocompromised cats may cause severe disease, and even death [10].

The global prevalence of *T. gondii* seropositivity, based on meta-analyses, has been reported to be 35% and 32.9% in cats and humans, respectively [11,12]. Several studies have examined the seroprevalence of *T. gondii* in cats in various countries, and, based on the results, a wide geographical variation is evident. 

Because of the vital role of cats in human infection, the high seropositivity rate highlights the need for current and comprehensive data on the epidemiology of *T. gondii* infection in cats. Only limited seroepidemiological data are available on *T. gondii* infection of cats in Greece [13], and there is only one study investigating the risk factors for seropositivity [14]. The objectives of the present study were to determine the prevalence of seropositivity against *T. gondii* in different populations of cats (including client-owned cats, stray cats, and cats living in catteries) living in different regions of Greece and to assess risk factors for *T. gondii* seropositivity.

## 2. Materials and Methods

### 2.1. Ethics Approval

The study protocol was reviewed and approved by the Animal Ethics Committee of the Faculty of Veterinary Science, University of Thessaly (13/16-6-15). Handling of the animals was in compliance with the European Communities Council Directive 2010/63/EU and state laws.

### 2.2. Study Population

A total of 457 cats from four different geographic areas of Greece (Attica, Thessaly, Crete, and Macedonia) were prospectively enrolled, between November 2013 and November 2016, as part of a previous study [15], and they were divided into three groups: client-owned cats, stray cats, and cats living in breeding catteries. These cats were presented by their owners (client-owned or cattery cats) or by two cat-rescue groups (stray cats) for wellness examination, vaccination, neutering, and/or medical treatment. Stray cats often originated from cat-dense environments (i.e., rescue colonies), and their age was estimated based on body size; dentition, including dental attrition (incisors, canines, premolars and molars); and other physical characteristics. [16,17,18]. Regarding physical characteristics, typical age-related physical effects in healthy cats manifest as changes in behavior, appearance, and daily function. 

The sampling size was calculated using the Epi Info 7 software (Centers of Disease Control and Prevention, Atlanta, Georgia) with a confidence interval of 95%, expected prevalence of 50%, and maximum error acceptable of 5%. The minimum number of systematically sampled cats was 384 cats.

Inclusion criteria included: a) body weight of ≥0.5 kg and b) an informed consent form signed by the owner or the rescuer. The cats that fulfilled the above two criteria were enrolled on a sequential basis, regardless of their health status.

Signalment and historical data were collected using a standardized questionnaire, and a thorough physical examination was performed for each cat. Historical information included: (a) signalment; (b) geographic origin and travel history; (c) prior and current ownership; (d) living conditions (indoors, outdoors); (e) diet (dry food, canned food, home cooked food, raw meat); (f) vaccination status (vaccinated, unvaccinated); (g) parasite prevention status (use of ectoparasiticides, non-use); (h) prior medical problems; (i) previous treatments; (j) chief presenting complaint; and (k) present health status (e.g., weight loss, reduced appetite, fecal characteristics).

### 2.3. Sample Collection and Laboratory Analyses

A total of 5 mL of blood was collected by jugular venipuncture from adult cats and 3.5 mL from young kittens. One milliliter of blood was sequentially transferred into two ethylenediaminetetraacetic acid (EDTA)-anticoagulated tubes (Sarstedt, Nümbrecht, Germany). The remaining blood was transferred in an anticoagulant-free tube, and the serum was harvested following centrifugation at 3000 rpm for 20 min. The serum aliquots were stored at −80 °C until indirect fluorescent antibody test (IFAT) for anti-*T. gondii* IgG was performed.

Anti-*T. gondii* IgG antibodies were detected using a commercially available IFAT kit (Biopronix Product Line, Agrolabo S.p.a., Metropolitan City of Turin, Italy) according to the manufacturer’s instructions using slides prepared with *Toxoplasma* tachyzoitis fixed on the wells. All sera were diluted 1:50 in phosphate-buffered saline (PBS, pH 7.2) for all untested sera and incubated on wells of the slides at 37 °C in a humidity chamber (Thermo Fisher Scientific, Marietta, OH, USA) for 30 minutes. The slides were rinsed twice in PBS, once in distilled water, and air-dried. Each well of the slides was probed with fluorescence-labelled anti-feline IgG (conjugate), ready for use (Biopronix Product Line, Agrolabo S.p.a., Metropolitan City of Turin, Italy) and incubated at 37 °C in a humidity chamber for 30 minutes. The slides were washed twice in PBS, once in distilled water, and dried as described above. Mounting fluid was added to each slide, and a coverslip was placed over the slide, carefully removing air bubbles caught under the coverslip, and all slides were examined using fluorescence microscopy (Olympus, Tokyo, Japan) at 400× magnification. Sera were screened starting from 1:50 dilution, and any serum sample positive at that dilution was titrated in serial twofold dilutions to the endpoint titer [19]. For each assay run, negative and positive controls that contained the IFAT kit were included. The size, appearance, and density of the characteristic fluorescence were compared with the positive and negative control reactions. Patterns of reactivity different than that seen in the positive control were considered nonspecific. 

### 2.4. Statistical Analysis

For univariate analysis, categorical data, regarding signalment and historical information, were compared between *T. gondii* seropositive and seronegative cats using Pearson’s χ^2^ or Fischer’s exact tests. The normality of the distribution of the continuous variables was tested using the Kolmogorov–Smirnov test. Normally distributed data are presented as means ± standard deviation and were compared between seropositive cats and seronegative cats using independent sample t-tests. Not normally distributed data are presented as medians and ranges and were compared between seropositive cats and seronegative cats using Mann–Whitney U tests. 

Variables that, in the univariate analysis, were different at 25% level of significance, between seropositive and seronegative cats, were selected as candidates for an initial logistic regression model. The initial model was subsequently reduced in a stepwise manner until only significantly different (*p* < 0.05) variables remained. Odds ratios (OR) derived from the reduced model were interpreted as measures of the risk of seropositivity. 

The analyses were performed using Stata 13 (Stata Corp, College Station, TX, USA) and SPSS 23 for Windows (IBM Corp, Armonk, NY, USA).

## 3. Results

A total of 457 cats were prospectively enrolled in the study. The age of these cats ranged from 6 weeks to 17 years (median: 2 years). Two hundred forty-two cats (53%) were males (86 neutered; 35.5% of the male cats), and 215 (47%) were females (52 neutered; 24.2% of the female cats). Fifteen cats (3.3%) were purebred, 427 (93.4%) were crossbred, and for 15 (3.3%) cats the breed was not recorded. Ninety-nine cats (21.7%) lived exclusively indoors, 336 (73.5%) outdoors, and for 22 cats (4.8%) living status was not recorded. Two hundred fifty-eight cats (56.5%) were living in Attica, 73 (16%) in Thessaly, 79 (17.3%) in Crete, and 47 (10.3%) in Macedonia. Two hundred sixty-nine cats (58.9%) were client-owned, 158 (34.6%) were stray, 21 (4.6%) were living in catteries, and for nine cats (2%) current ownership was not recorded. Three hundred sixty-six cats (80.1%) lived in urban areas and 68 (14.9%) in rural areas, while for 23 (5%) cats their habitat was not recorded. Two hundred sixty cats (56.9%) were adopted as strays, while 102 (22.3%) were adopted as nonstray cats, including 75 (16.4%) client-owned cats, 24 (5.3%) cats living in catteries, and three (0.7%) cats living in pet shops. For 95 cats (20.7%), the acquisition was not recorded. Three hundred forty-four cats (75.3%) came in contact with other cats either for a long period (constant contact, such as multicat households) or occasionally (such as rescue colonies) and 56 (12.3%) were not in contact with other cats, while for 57 cats (12.5%) contact with other cats was not recorded. Of the cats who came into contact with other cats, 57 (21.1%) were in contact with 1–2 cats, 11 (4.1%) were in contact with 3 cats, and 202 (74.8%) were in contact with more than 3 cats. Seven cats (10.3%) had a history of cat-bite wounds and 154 (33.7%) had no such a history, and for 256 (56%) the history of cat bite was not recorded. One hundred sixty-three cats (35.7%) were vaccinated against calicivirus, herpesvirus-1, panleukopenia, and rabies (CHPR) and 175 (38.3%) were unvaccinated, while for 119 cats (26%) the vaccination status was not recorded. One hundred fifty-nine cats (34.8%) had been treated with an ectoparasiticide and 108 (23.6%) had not been on preventative ectoparasiticide, while for 190 cats (41.6%) the preventative ectoparasiticide status was not recorded. Ninety cats (19.7%) were infested with fleas and 356 (77.9%) were not infested with fleas, while for 11 cats (2.4%) flea infestation status was not recorded. Seven cats (1.5%) were infested with ticks and 438 (95.9%) were not infested with ticks, while for 12 cats (2.6%) tick infestation status was not recorded. Ninety-four cats (20.6%) consumed prey, raw meat, and/or unpasteurized milk and 118 cats (25.8%) did not consume the above, while for 245 cats (53.6%) the consumption of prey, raw meat, and/or unpasteurized milk was not recorded. 

Of the 457 cats, a total of 95 (20.8%) were seropositive for *T. gondii*. The univariate associations between seropositivity and signalment or historical data are presented in Table 1.

Multivariate analysis indicated three factors that were independently associated with *T. gondii* seropositivity (Table 2). *Toxoplasma gondii*-seropositive cats were older, more likely to have history of cat-fight trauma, and more likely to be unvaccinated against CHPR, compared to seronegative cats.

## 4. Discussion

This is the first study investigating risk factors for *T. gondii* seropositivity in different populations of cats in Greece, including client-owned cats, stray cats, and cats who lived in catteries.

The seroprevalence identified here is similar to that reported in a previous prospective study in Greece, where it was reported to be 21.8% [13,14]. However, in that study only stray cats were included. In addition, the seroprevalence in our study is similar to that reported in other countries, including Italy, Portugal, United Kingdom, the Netherlands, Argentina, Brazil, and China [20,21,22,23,24,25,26]. Globally, studies evaluating the seroprevalence for *T. gondii* have shown a wide variation. In Europe, seroprevalence has been reported to be 41.0–60.8% in Northern Europe [27,28], 10.0–84.7% in Southern Europe [29,30], 19.2–65.5% in Western Europe [26,31], 14.7–39.3% in Eastern Europe [32,33], and 47.0–81.3% in Central Europe [32,34]. In America, studies have reported an overall seroprevalence of 1.1–100% in North America [35,36], 0–82.8% in South America, and 25% in Central America [37,38,39]. In addition, the reported seroprevalence in North Africa varies from 50% to 97.5% in North Africa and from 4.4% to 36.2% in West Africa, and it is reported to be 3.9% in South Africa [40,41,42,43,44], while in Asia it ranges between 2.2% and 82.8% [45,46]. This tremendous variability may reflect differences in the demographics and the geographic origin of the cats, the accuracy of the diagnostic tests, and/or the overtime changes in the prevalence of these infections. The greatest risk factor for *T. gondii* seropositivity is hunting and ingestion of tissue cysts in prey species, including rodents and birds. Additionally, ingestion of mechanical vectors such as cockroaches and earthworms has been suggested as a possible mode of infection. Our study showed that cats with a history of cat-bite wounds were more likely to be *T. gondii*-seropositive than cats with no such history. To our knowledge, this is the first time that a significant association between cat-bite wounds and seropositivity to *T. gondii* has been reported. The major route of transmission among cats is the ingestion of bradyzoites within tissue cysts. However, transmission through bite wounds that introduce saliva containing tachyzoites may also occur [47]. In this case, the biting cat must be in the acute phase of infection, in order to have tachyzoites in blood (parasitemia) and body secretions [3,48,49]. However, cats with history of cat-bite wounds are more likely to live outside and thus to hunt and eat intermediate hosts, which is a well-established risk factor for *T. gondii* seropositivity. Therefore, we suggest that the association between cat-bite wounds and seropositivity most likely reflects the consumption of the intermediate hosts by the cats. It is worth mentioning that based on the results of the univariate analysis, cats consuming prey, raw meat, and/or unpasteurized milk had a statistically significant higher risk of acquiring *T. gondii* than cats that did not consume any raw food. This finding is in accordance with the results of several other studies [22,50,51,52,53,54], because cats consuming raw meat may have a higher chance of ingesting tissue cysts containing bradyzoites. However, consumption of prey, raw meat, and/or unpasteurized milk was not statistically significant in the multivariate model.

In our study, *T. gondii*-seropositive cats were significantly older than *T. gondii*-seronegative cats. This finding is consistent with previous studies, and it may be explained by the fact that older cats have more time to be exposed to the parasite [50,55,56] and not because older cats may have a weaker immune response, which can make them more susceptible to *T. gondii* infection and more likely to develop antibodies against the parasite. Although seropositivity increases with age, indicating postnatal transmission of *T. gondii*, in a study published in 2014, the authors showed a significantly higher seroprevalence of *T. gondii* in young cats aged 2 months (100.0%) compared to adult cats (66.8%) [57]. This finding probably was due to the transfer of IgG maternal antibodies through the colostrum [50]. It is known that maternally transferred antibodies disappear in the cat by 12 weeks of age.

Lack of vaccination against CHPR was found to be an independent risk factor for *T. gondii* seropositivity. This is consistent with the results of a recent study on the epidemiology of *T. gondii* in 155 cats from Cyprus and, presumably, reflects the better quality of life and veterinary care of the vaccinated cats compared to unvaccinated ones. Unvaccinated cats have an increased chance of becoming infected by calicivirus, herpesvirus-1, and/or panleukopenia virus, and to develop clinical illness. Concomitant illnesses render cats more susceptible to *T. gondii* infection. Furthermore, they may lead to reactivation of tissue cysts in chronically infected but seronegative cats, with subsequent release of bradyzoites and seroconversion [58]. Finally, unvaccinated cats are more likely to be stray and thus to be more prone to eating intermediate hosts.

With regards to gender, male sex has been considered a risk factor for *T. gondii* seropositivity. However, in our study based on the results of univariate analysis, females were significantly more likely to be *T.gondii*-seropositive compared to male cats. Our finding is consistent with other studies that showed higher seropositivity in female cats and could be explained by the fact that females behave differently than males and may roam more based on the season or hormonal imbalances [53,57,59,60]. However, female gender was not statistically significant in the multivariate model.

To our knowledge, there is no study showing that body weight could be a risk factor for *T. gondii* seropositivity. In this context, in the present study, body weight was not significantly higher in *T. gondii*-seropositive cats compared to seronegative cats, despite the higher seropositivity rate in cats who weighed more. Because body weight increases during growth, and because multivariate analysis showed that older age was associated with increased risk of *T. gondii* seropositivity, this higher seropositivity rate most likely reflects the fact that more seropositive cats were adults (and thus fully grown) compared to seronegative cats. 

Our study, based on the results of the univariate analysis, showed that cats with outdoor access were significantly more likely to be *T. gondii*-seropositive compared to cats living strictly indoors, and this is in agreement with several previous reports [22,52,61,62]. Cats living outdoors or having outdoor access are more likely to hunt and eat intermediate hosts, who might be infected, compared to cats that live indoors and typically eat safe processed food [43,63,64]. Furthermore, outdoors cats have an increased chance of becoming infected by drinking oocyst-contaminated water [65]. However, outdoor access was not statistically significant in the multivariate model.

Living in rural areas is consider a risk factor for *T. gondii* seropositivity in cats [66,67]. This is because rural areas have a higher prevalence of *T. gondii* infection in the environment due to factors such as a higher density of livestock, increased exposure to soil, and greater likelihood of hunting and consuming rodents and other prey [28,52,59,68]. However, in the present study, the risk of seropositivity was not significantly different in cats living in rural areas than in cats living in urban areas, despite the higher seropositivity rate in cats living in rural areas. It is possible that living in rural areas may not always be a risk factor for *T. gondii* seropositivity in cats, depending on various circumstances. For example, if a cat living in a rural area has no access to the outdoors and is kept exclusively indoors, its risk of exposure to *T. gondii* oocysts in the environment is significantly reduced. Similarly, if the cat is fed a commercially prepared diet that does not include raw or undercooked meat, its risk of acquiring *T. gondii* from infected prey is also lower. In addition, another explanation could be the fact that the prevalence of *T. gondii* in the soil can vary by location, and cats in rural areas may have less access to contaminated soil than cats in urban or suburban areas. Similar to our study, a few studies found no significant difference in the risk of seropositivity between cats living in rural areas and urban areas [69].

In our study, based on both univariate and multivariate logistic regression analysis, cats who came in contact with other cats either for a long period (constant contact, such as multicat households) or occasionally (such as rescue colonies) were not significantly more likely to be seropositive compared to cats who were not in contact with other cats. This finding is not surprising. Most cats are infected by ingesting intermediate hosts (or infected animal tissues) and shed resistant oocysts with their feces. Cats could be infected through the fecal–oral route by indirect transmission, such as contaminated litter trays or brushes and through mutual grooming, such as when one infected cat licks another. Regarding litter boxes, infected cats passed unsporulated (noninfectious) oocysts into the environment, and once passed to the environment, sporulation occurs in 1–5 days. Often, litter trays are cleaned daily so that oocysts do not have sufficient time to sporulate. Regarding grooming, even when cats are shedding oocysts in their feces, oocysts cannot be found on their coat [6]. In addition, studies in dogs have shown that oocysts do not sporulate on their fur, and the same might be true for cats [70].

Flea infestation is not directly a risk factor for *T. gondii* seropositivity in cats. Fleas can transmit other diseases and parasites to cats (e.g. *Bartonella henselae*), but they are not known to transmit *T. gondii*. However, in our study, based on the results of the univariate analysis, cats infested with fleas were significantly more likely to be *T. gondii*-seropositive compared to cats without flea infestation. Accordingly, in the present study, the seropositivity rate was higher in cats that had not been on a preventative ectoparasiticide than cats previously treated with an ectoparasiticide, although in the multivariate analysis this association was not statistically significant. This finding is consistent with the results of a recent study conducted in 2021, where lack of ectoparasite prevention was not found to be a risk factor for *T. gondii* seropositivity [69]. Cats infested with fleas are more likely to live outside and thus to hunt and eat intermediate hosts, who might be infected. However, flea infestation was not statistically significant in the multivariate model.

## 5. Conclusions

This study shows a high prevalence of seropositivity for *T. gondii* among cats in Greece. Thus, feline toxoplasmosis should be considered a major public health concern in this country, necessitating the establishment of optimal prevention strategies, taking into consideration the high-risk groups of cats identified in this study, i.e., older cats, cats with a history of cat-fight trauma, and cats unvaccinated against calicivirus, herpesvirus-1, panleukopenia, and rabies. 

Future prospective studies in naturally infected cats are warranted in order to determine the prevalence of feline toxoplasmosis in Greece and assess the risk factors for the appearance of the disease.

## Figures and Tables

**Table 1:** Univariable associations between seropositivity for *Toxoplasma gondii* among 457 cats and the signalment and historical data that were collected using a standardized questionnaire.

		Anti-*T. gondii* IgG Antibodies				
Variables	Categories	Positive (%)	Negative (%)	Total	Missing Data	*p*-Value
Sex	Male	39 (16.1%)	203 (83.9%)	242	0	0.009 ^*^
	Female	56 (26.0%)	159 (74.0%)	215		
Neutering	Yes	26 (18.8%)	112 (81.2%)	138	6	0.535
	No	67 (21.4%)	246 (78.6%)	313		
Breed	Purebred	1 (6.7%)	14 (93.3%)	15	15	0.326
	Crossbreed	90 (21.1%)	337 (78.9%)	427		
Age (years) ^a^		2.5 (0.13–17)	1.05 (0.13–15)	434	23	<0.001 ^*^
Body weight (kg) ^b^		3.5 (0.85–7.2)	3.2 (0.5–9)	411	46	0.173 ^*^
Cat acquisition	Client-owned	14 (18.7%)	61 (81.3%)	75	95	0.625
	Stray	58 (22.3%)	202 (77.7%)	260		
	Cattery	3 (12.5%)	21 (87.5%)	24		
	Pet shop	0 (0.0%)	3 (100.0%)	3		
Current ownership	Client-owned	51(19.0%)	218 (81.0%)	269	9	0.433
	Stray	38 (24.0%)	120 (76.0%)	158		
	Cattery	4 (19.0%)	17 (81.0%)	21		
Living conditions	Indoors	9 (9.0%)	90 (91.0%)	99	22	0.001 ^*^
	Outdoors	81 (24.0%)	255 (76.0%)	336		
Living area	Urban	72 (19.7%)	294 (80.3%)	366	23	0.204 ^*^
	Rural	18 (26.5%)	50 (73.5%)	68		
Contact with other cats	Yes	72 (20.9%)	272 (79.1%)	344	57	0.249 ^*^
	No	8 (14.3%)	48 (85.7%)	56		
Number of in-contact cats	0 cats	8 (14.3%)	48 (85.7%)	56	131	0.669
	1–2 cats	9 (15.8%)	48 (84.2%)	57		
	3 cats	3 (27.3%)	8 (72.7%)	11		
	>3 cats	36 (17.8%)	166 (82.8%)	202		
History of cat-bite wounds	Yes	12 (25.5%)	35 (74.5%)	47	256	0.013 ^*^
	No	17 (11.0%)	137 (89.0%)	154		
Vaccinated against CHPR	Yes	25 (15.3%)	138 (84.7%)	163	119	0.01 ^*^
	No	47 (26.9%)	128 (73.1%)	175		
Use of ectoparasiticide	Yes	27 (17.0%)	132 (83.0%)	159	190	0.154 ^*^
	No	26 (24.1%)	82 (75.9%)	108		
Flea infestation	Yes	27 (30.0%)	63 (70.0%)	90	11	0.014 ^*^
	No	65 (18.3%)	291 (81.7%)	356		
Tick infestation	Yes	1 (14.3%)	6 (85.7%)	7	12	1
	No	91 (20.8%)	347 (79.2%)	438		
Consumption of prey, raw meat, and/or unpasteurized milk	Yes	24 (25.5%)	70 (74.5%)	94	245	0.002 ^*^
	No	11 (9.3%)	107 (90.7%)	118		

^*^ Variable that was used in the logistic regression model (*p*-value < 0.25). ^a^ Age was a continuous variable. ^b^ Body weight was a continuous variable. CHPR: calicivirus, herpesvirus-1, panleukopenia, rabies.

**Table 2:** Multivariate analysis of risk factors for *Toxoplasma gondii* seropositivity.

Variables	Odds Ratio	Confidence Interval	*p*-value
Age (years)	1.33	1.15–1.54	<0.001
History of cat-bite wounds	3.88	1.54–9.81	0.004
Vaccinated against CHPR	0.10	0.07–0.55	0.002

CHPR: calicivirus, herpesvirus-1, panleukopenia, rabies.

## Data Availability

The data presented in this study are available in Table 1 and Table 2.
